# Adjuvant Corticosteroids With Surgery for Chronic Subdural Hematoma: A Systematic Review and Meta-Analysis

**DOI:** 10.3389/fnins.2021.786513

**Published:** 2021-12-08

**Authors:** Min Shi, Ling-fei Xiao, Ting-bao Zhang, Qing-wen Tang, Wen-yuan Zhao

**Affiliations:** ^1^Department of Neurosurgery, Zhongnan Hospital of Wuhan University, Wuhan, China; ^2^Department of Orthopaedics, Zhongnan Hospital of Wuhan University, Wuhan, China

**Keywords:** corticosteroids, chronic subdural hematoma, adjuvant treatment, recurrence, mortality, poor outcome

## Abstract

The use of adjuvant corticosteroids with surgery for chronic subdural hematoma (CSDH) has received considerable attention in recent years. However, there is no conclusive evidence regarding its effectiveness and safety for CSDH. Therefore, we performed a meta-analysis and systematic review to evaluate the effectiveness and safety of corticosteroids as an adjuvant treatment for the treatment of CSDH. We comprehensively searched electronic databases (PubMed, EMBASE, Cochrane Library, and Web of Science) to identify relevant trials that investigated the efficacy and safety of adjuvant corticosteroids with surgery for CSDH, published from inception until May 2021. Outcome measures included recurrence rate, all-cause mortality, good functional outcome, length of hospitalization, and adverse events. We used the Cochrane risk of bias method to evaluate the quality of randomized controlled trials (RCTs), and the Newcastle Ottawa Scale to evaluate the quality of observational studies. We included nine studies, consisting of three RCTs and six observational studies, that compared corticosteroids as an adjuvant treatment to surgery with surgery alone. Pooled results revealed that the risk of recurrence was significantly reduced in patients who received adjuvant corticosteroids with surgery compared to those who underwent surgery alone (relative risk [RR] = 0.52, 95% confidence interval [CI] = 0.39–0.69, *p* < 0.00001). However, no statistically significant difference was observed between these groups in all-cause mortality (RR = 0.91, 95% CI = 0.37–2.23, *p* = 0.83), good functional outcome (RR = 1.03, 95% CI = 0.96–1.10, *p* = 0.47), length of hospitalization (MD = 0.35, 95% CI = –2.23 to 1.67, *p* = 0.83), and infection rates (RR = 0.99, 95% CI = 0.64–1.53, *p* = 0.95). Adjuvant corticosteroids with surgery reduce the risk of recurrence of CDSH, but do not improve the all-cause mortality or functional outcome, as compared to surgery alone. These findings support the use of adjuvant corticosteroids with surgery for CSDH patients. Further high-quality RCTs are required to confirm the efficacy and safety of adjuvant corticosteroids in the treatment of CSDH patients.

## Introduction

Chronic subdural hematoma (CSDH) is one of the most common neurosurgical conditions, characterized by relatively slow abnormal accumulation of blood and fluid in the subdural space (Cenic et al., 2005; Santarius et al., 2010; Kolias et al., 2014). The incidence of CSDH is approximately 15/100,000/year in Western societies; and it is higher in the older population (Santarius et al., 2008; Yang and Huang, 2017). In recent decades, the aging population and increasing use of anticoagulants and antiplatelets has gradually increased the incidence of CSDH globally (Rauhala et al., 2019; Hutchinson et al., 2020). However, to date, there is no consensus on the standard treatment for CSDH. The most commonly accepted treatment for symptomatic CSDH is surgical evacuation of the hematoma, mainly by burr hole drainage, to eliminate the space-occupying effects of the hematoma (Liu et al., 2014; Manickam et al., 2016; Toi et al., 2018; Yuan et al., 2018). Although most patients have a good prognosis with surgery, complications and adverse events may still occur (Schmidt et al., 2015). The recurrence rates after surgery are 5–30% (Ryu et al., 2018), and poor functional outcome occurs in up to 22% of patients (Brennan et al., 2017). Therefore, many studies have been conducted to identify better treatment options for CSDH.

The mechanism of CSDH formation is unclear, but an increasing number of studies suggest that inflammation is involved in the disease process and propagation by promoting self-sustained neoangiogenesis and fibrinolysis (Edlmann et al., 2017; Holl et al., 2018). Based on these findings, numerous adjuvant therapies with surgery have been investigated, including corticosteroids, atorvastatin, and middle meningeal artery embolization (Berghauser Pont et al., 2012; Jiang et al., 2018; Catapano et al., 2020). Corticosteroids reduce CSDH growth by inhibiting inflammation, reducing vascular permeability, and preventing the abnormal accumulation of fluid in the subdural space (Frati et al., 2004; Stanisic et al., 2012a; Edlmann et al., 2017). Therefore, corticosteroids are commonly used as monotherapy or adjuvant treatment for CSDH (Laldjising et al., 2020). Previous studies suggested that compared to surgery alone, corticosteroids alone may avoid the need for surgery, and adjunctive corticosteroids with surgery may reduce the risk of recurrence in CSDH patients (Yadav et al., 2016; Guo et al., 2020). However, some studies reported lack of improvement, or even worsening, in outcomes of CSDH with corticosteroid use (Hutchinson et al., 2020). Therefore, we performed this meta-analysis to evaluate the efficacy and safety of adjuvant corticosteroids with surgery in CSDH patients.

## Materials and Methods

We performed the current study according to the Preferred Reporting Items for Systematic Reviews and Meta-Analyses (PRISMA) guidelines. The PRISMA checklist is presented in Supplementary File 1.

### Literature Search

We comprehensively searched PubMed, EMBASE, Cochrane Library, and Web of Science for relevant articles published until May 2021. Articles were searched using the following terms: (corticosteroid or steroid or prednisolone or prednisone or dexamethasone or cortisol or hydrocortisone or glucocorticoid or methylprednisolone) and (subdural hematoma or subdural hemorrhage or CSDH). The free terms are presented in Supplementary File 2.

### Selection Criteria

We included randomized controlled trials (RCTs) and observational studies that included at least 20 adult patients (age ≥ 18 years) with CSDH, and compared the effects of adjuvant corticosteroids with surgery and surgery alone (more than 90% treated with surgery in both experimental groups and in control groups) on at least one relevant outcome measure.

We excluded studies if they did not report any relevant outcome measures or included children (age < 18 years). We also excluded review articles, conference reports, systematic reviews, and case reports, as well as studies without a control group.

### Outcome Measures

The primary outcome was the recurrence rate at the longest follow-up. The secondary outcomes included all-cause mortality, good functional outcome, length of hospitalization, and adverse events at the longest follow-up. We attempted to evaluate some other outcomes (such as hematoma size evolution), but we were unable to identify adequate data from the included studies.

Recurrence rate was defined as clinical or radiological recurrence requiring repeat intervention. All-cause mortality was defined as death from any possible cause at the longest follow-up. Good neurological function outcome was defined as Glasgow Outcome Score (GOS) ≥ 4, modified Rankin Scale (mRS) score ≤ 2, or Markwalder Grading Scale (MGS) score 0–1.

### Data Extraction and Quality Assessment

Two investigators (MS and LX) independently searched and reviewed the full text reports of all eligible studies. The following information was extracted, using a pre-defined standardized form: name of first author, publication year and country, study period and design, sample size, sex, age, follow-up duration, outcome measures, and intervention details. Disagreements were resolved through discussion with a third investigator (TZ).

We assessed the quality of each included study. For RCTs, we used the criteria outlined in the Cochrane Handbook, which includes seven domains (random sequence generation; allocation concealment; blinding of participants and personnel; blinding of outcome assessment; incomplete outcome data; selective outcome reporting; and other bias) (Supplementary File 3). For observational studies, we used the Newcastle Ottawa Scale for quality assessment. Disagreements in the process of quality assessment were resolved by discussion or by a third investigator (TZ) (Table 1).

**TABLE 1:** Quality assessment by the Newcastle Ottawa Scale for cohort studies.

Study	Selection				Comparability	Outcome			Scores
	A	B	C	D	E	F	G	H	
Lodewijkx et al. (2021)	1	1	1	0	2	1	1	1	8
Fountas et al. (2019)	1	1	1	0	1	1	1	1	7
Qian et al. (2017)	1	1	1	0	1	1	1	1	7
Delgado-López et al. (2009)	1	1	1	0	0	1	1	1	6
Dran et al. (2007)	1	1	1	0	1	1	1	1	7
Sun et al. (2005)	1	1	1	1	1	1	1	1	8

*A, Representativeness of exposed cohort; B, Selection of non-exposed cohort; C, Ascertainment of exposure; D, Outcome of interest not present at start; E, Comparability main or Comparability additional factors; F, Assessment of outcome; G, Follow-up long enough; H, Adequacy of follow up.*

### Statistical Analysis

We performed the data synthesis and analysis using the Review Manager software (RevMan; The Nordic Cochrane Centre, Copenhagen, Denmark). We calculated the pooled risk ratios (RRs) and corresponding 95% confidence intervals (CIs), and displayed the results as forest plots. The RCTs and observational studies were analyzed together as well as separately. Heterogeneity among the studies was evaluated using the I^2^ statistic and Cochrane Q-statistic test. If I^2^ was > 50% or p was < 0.10 (i.e., there was significant heterogeneity), we used the random-effects model. Otherwise, we used the fixed-effects model. We performed subgroup and sensitivity analyses to identify and minimize heterogeneity. Publication bias of the primary outcome was evaluated using funnel plots.

## Results

### Study Selection

**FIGURE 1:**
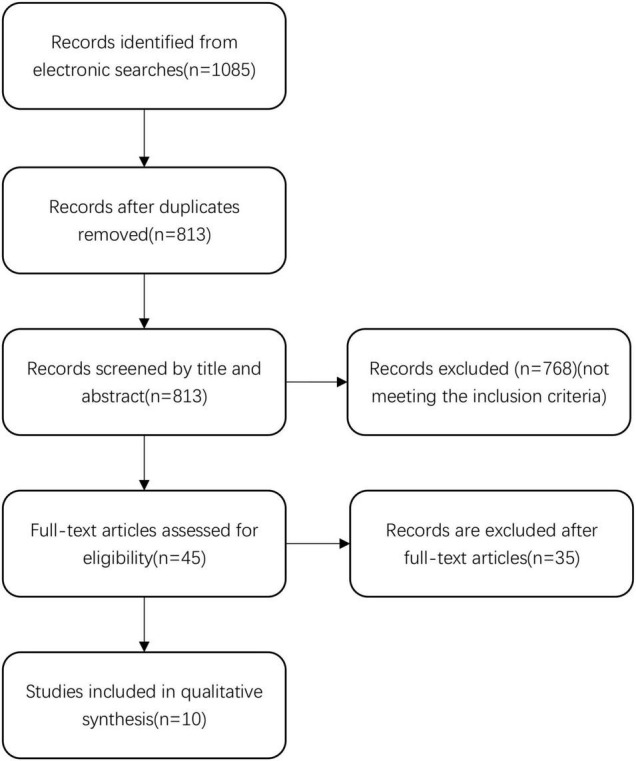
Flow diagram of the study selection process.

We identified 1,085 records in the initial search. After removing 272 duplicate records, we screened the title and abstract of the remaining 813 records. We excluded 768 records that did not meet the inclusion criteria, and reviewed the full text reports of the remaining 45 records. After the full-text review, we excluded 35 records. Finally, 10 articles were selected for inclusion in the final analysis (Sun et al., 2005; Dran et al., 2007; Delgado-López et al., 2009; Chan et al., 2015; Qian et al., 2017; Fountas et al., 2019; Mebberson et al., 2020; Lodewijkx et al., 2021; Ng et al., 2021). Details of the literature search and review are displayed in Figure 1.

### Basic Characteristics of Included Studies

The basic characteristics of the included studies are presented in Table 2. We included 10 studies consisting of 2,568 patients. Of the 10 included studies, four were RCTs (Chan et al., 2015; Hutchinson et al., 2020; Mebberson et al., 2020; Ng et al., 2021), five were retrospective cohort studies (Dran et al., 2007; Delgado-López et al., 2009; Qian et al., 2017; Fountas et al., 2019; Lodewijkx et al., 2021), and one was a prospective cohort study (Sun et al., 2005). Three studies were conducted in China, six in Europe, and one in Australia. Most studies were published after 2015 (*n* = 7). The studies included 47–748 participants, aged 66.3–78 years, who were followed for 3–17.5 months. The dosage of corticosteroids varied considerably between studies, with the first dose ranging from 12 to 24 mg/day, and the total dosage of corticosteroids ranged from 90 to 388 mg.

**TABLE 2:** Baseline characteristic of included studies.

Study	Country	Design	Duration	N	TP/CP	M/F	Age	Outcome	FUP	Experimental interventional
Lodewijkx et al. (2021)	Netherlands	R	2010–2015	525	278/247	393/132	74 ± 12	RR/mortality/adverse events	6 m	DXM:16 mg/day
Ng et al. (2021)	France	RCT	NR	155	78/77	114/41	D:75.6 ± 10.6 C:72.7 ± 15.0	RR/mortality/good outcome/adverse events	12 m	Prednisone: an initial dose of 1 mg/kg/day for 7 days, with slow tapering
Hutchinson et al. (2020)	United Kingdom	RCT	2015–2019	748	375/373	554/194	D:74.5 ± 11.8 C:74.3 ± 11.0	RR/mortality/good outcome/LH/adverse events	6 m	DXM: an initial dose of 16 mg/day for 3 days, with slow tapering
Mebberson et al. (2020)	Australia	RCT	2014–2018	47	23/24	34/13	D:73.39 ± 15.4 C:75.13 ± 15.5	RR/LH/mortality/good outcome/adverse events	6 m	DXM: an initial dose of 16 mg/day for 3 days, with slow tapering
Fountas et al. (2019)	Greece	R	2012–2016	171	24/137	120/51	76.4 ± 9.3	RR/mortality/LH	> 3 m	DXM: 24 mg/day for 1 week, with slow tapering
Qian et al. (2017)	China	R	2010–2015	242	75/167	148/94	66.3(36–93)	RR	6 m	DXM:13.5 mg/day, with slow tapering
Chan et al. (2015)	China	RCT	2000–2006	248	122/126	177/71	71.3	RR/mortality/good outcome/adverse events	6 m	DXM: an initial dose of 16 mg/day for 4 days, with slow tapering
Delgado-López et al. (2009)	Spain	R	2001–2006	122	25/19	84/38	78(25–97)	RR/mortality/good outcome/adverse events	25 w	DXM:12 mg/day, with slow tapering
Dran et al. (2007)	France	R	1998–2002	198	142/56	142/56	75 ± 13	Mortality/LH/adverse events	17.5 m	Methylprednisolone: 0.5 mg/kg/day for 1 month
Sun et al. (2005)	China	P	1998–1999	112	69/13	64/48	75(39–91)	RR/mortality/good outcome/LH/adverse events	6 m	DXM: 16 mg/day for 21 days

*TP, treatment group; CP, control group; M, male; F, female; FUP, follow-up period; R, retrospective; RCT, randomized controlled trial; P, prospective study; RR, recurrence rate; m:month; DXM, dexamethasone; NR; not reported; LH, length of hospitalization.*

### Recurrence Rate

**FIGURE 2:**
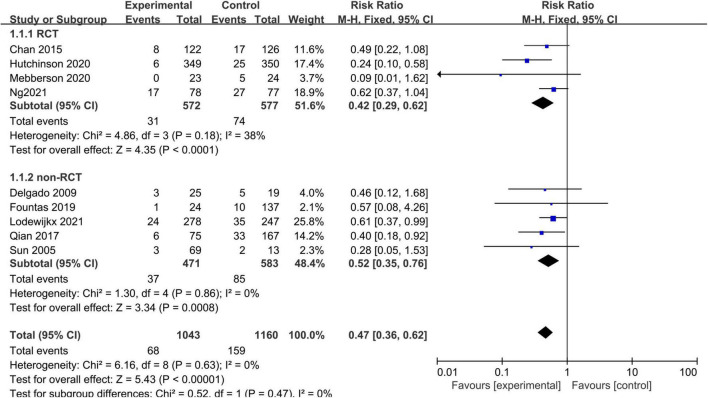
Meta-analysis of adjuvant corticosteroid group compared to the surgery alone group on recurrence rate.

Nine studies, consisting of 2,203 patients, provided data on CSDH recurrence that required re-intervention (Figure 2). A fixed-effects model was used because of a lack of significant heterogeneity between studies (I^2^ = 0%, *p* = 0.63). The pooled results showed that CDSH recurrence rate was lower in the adjuvant corticosteroid group compared to the surgery alone group (RR = 0.47, 95% CI = 0.36–0.62, *p* < 0.00001). Subgroup analysis by study design showed the recurrence rates were lower for the adjuvant corticosteroid group compared to the surgery alone group in RCTs (RR = 0.42, 95% CI = 0.29–0.62, *p* < 0.0001) and observational studies (RR = 0.52, 95% CI = 0.35–0.76, *p* = 0.0008).

### All-Cause Mortality

**FIGURE 3:**
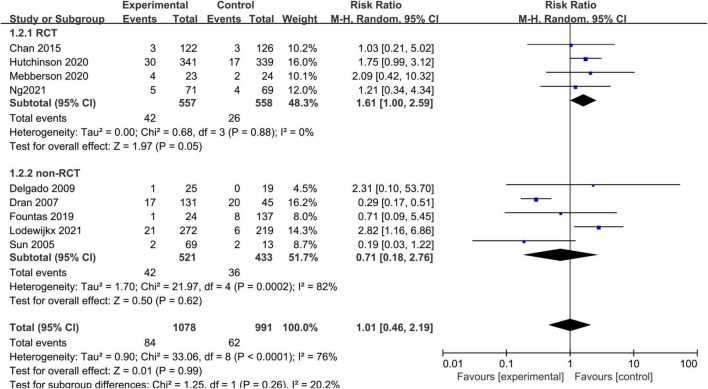
Meta-analysis of adjuvant corticosteroid group compared to the surgery alone group on all-cause mortality.

Nine studies, consisting of 2,069 patients, compared the all-cause mortality between the adjuvant corticosteroid group and surgery alone group. We used the random-effects model because of significant heterogeneity (I^2^ = 76%, *p* < 0.0001). The pooled results showed that the all-cause mortality rate was not significantly different between the adjuvant corticosteroid group and surgery alone group (RR = 1.01, 95% CI = 0.46–2.19, *p* = 0.99) (Figure 3). The subgroup analysis by study type showed that there were no statistically significant differences all-cause mortality between the adjuvant corticosteroid group and surgery alone group in RCTs (RR = 1.61, 95% CI = 1.00–2.59, *p* = 0.05) or observational studies (RR = 0.71, 95% CI = 0.18–2.76, *p* = 0.62). Meanwhile, subgroup analysis showed that the study design may be the potential source of heterogeneity.

### Good Functional Outcome

**FIGURE 4:**
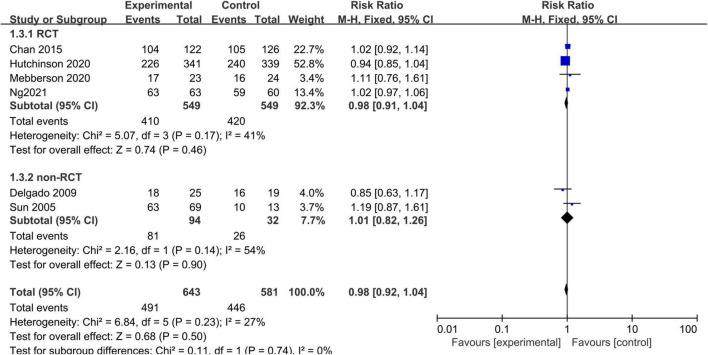
Meta-analysis of adjuvant corticosteroid group compared to the surgery alone group on good functional outcome.

Six studies, consisting of 1,224 patients, reported data on good neurological functional outcome. There was no significant heterogeneity (I^2^ = 27%, *p* = 0.23) between the studies so a fixed-effects model was used. The pooled results showed that there was no significant difference in the rate of good functional outcome between the adjuvant corticosteroid group and surgery alone group (RR = 0.98, 95% CI = 0.92–1.04, *p* = 0.50) (Figure 4). Subgroup analysis by study type showed no statistically significant differences in the rates of good functional outcome between the adjuvant corticosteroid group and surgery alone group in RCTs (RR = 0.98, 95% CI = 0.91–1.04, *p* = 0.46) or observational studies (RR = 1.01, 95% CI = 0.82–1.26, *p* = 0.90).

### Length of Hospitalization

**FIGURE 5:**
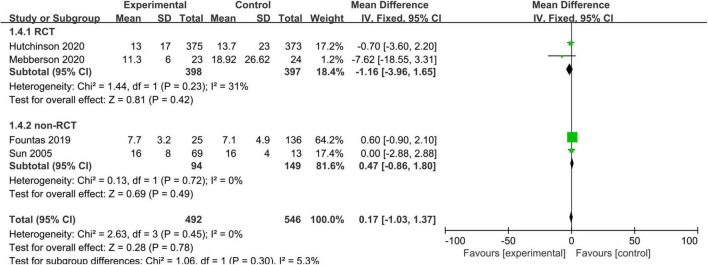
Meta-analysis of adjuvant corticosteroid group compared to the surgery alone group on length of hospitalization.

Three studies, consisting of 290 patients, reported the length of hospitalization. We used a fixed-effects model due to no substantial heterogeneity among the studies (I^2^ = 0%, *p* = 0.45). The pooled results showed no statistically significant difference in the length of hospitalization between the adjuvant corticosteroid group and surgery alone group (MD = 0.17, 95% CI = –1.03 to 1.37, *p* = 0.78) (Figure 5).

### Adverse Events

**FIGURE 6:**
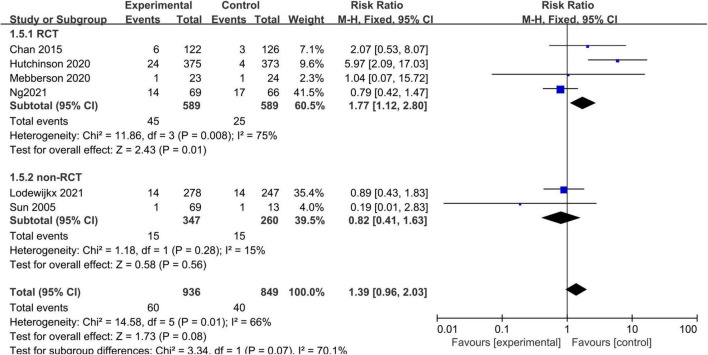
Meta-analysis of adjuvant corticosteroid group compared to the surgery alone group on infections.

Some adverse events were not reported consistently across the included studies. Therefore, we only analyzed certain important adverse events (such as infection rates) with adequate data. Six studies, consisting of 1,785 patients, reported infections. The pooled results showed no statistically significant difference in infections between the adjuvant corticosteroid group and surgery alone group (RR = 1.39, 95% CI = 0.96–2.03, *p* = 0.08) (Figure 6).

### Publication Bias

**FIGURE 7:**
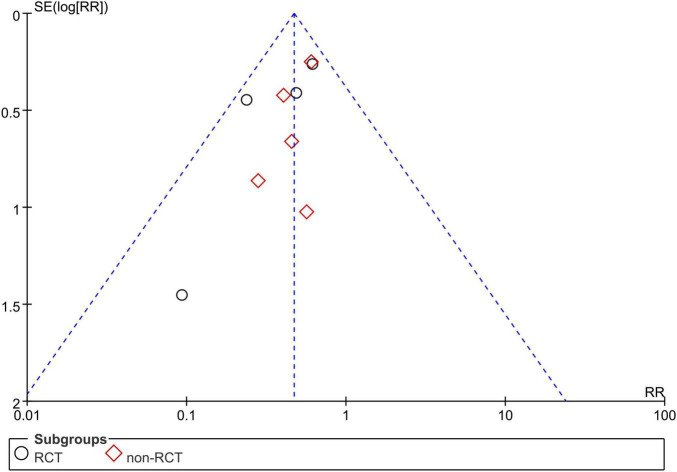
Funnel plot of publication bias of recurrence rate.

Due to the limited number of studies, we only assessed publication bias for studies reporting the recurrence rate. There was no obvious publication bias in the current meta-analysis (Figure 7).

## Discussion

In this meta-analysis and systematic review, we evaluated the efficacy and safety of adjuvant corticosteroids with surgery for the treatment of CSDH. Our results showed that the recurrence rate was significantly lower for patients who received adjuvant corticosteroids with surgery compared to those who underwent surgery alone. However, adjuvant corticosteroids did not significantly improve the all-cause mortality, functional outcome, or length of hospitalization. Adjuvant corticosteroids did not increase the risk of important adverse events.

CSDH is one of the most common neurosurgical conditions, especially in elderly patients (Adhiyaman et al., 2002). Surgical removal of CSDH is expected to become the most common neurosurgical surgery among adults by 2030 in the United States (Holl et al., 2018). Although surgical removal of the hematoma remains the most effective treatment approach, the incidence of postoperative recurrence of hematoma is high (Jung et al., 2015; Schmidt et al., 2015). Therefore, more effective treatments are required that decrease the recurrence rate and improve the prognosis. Several other treatments have been proposed to improve the management of CSDH patients. Ban et al. (2018) reported that middle meningeal artery embolization leads to better outcomes after CSDH compared to conventional management. He et al. (2021) reported that atorvastatin improves the prognosis of CSDH patients. Holl et al. (2019) demonstrated that adjuvant corticosteroids with surgery may be effective in the treatment of CSDH.

Although the exact mechanism of CSDH formation is unknown, studies demonstrated that pathological angiogenesis and inflammation are involved (Edlmann et al., 2017). Mediators, such as angiopoietins and matrix metalloproteinases, play an important role in angiogenesis that contributes to CSDH formation (Edlmann et al., 2017). Inflammatory cells, including neutrophils, lymphocytes, macrophages, and eosinophils, mediate the process of inflammation (Stanisic et al., 2012b). Based on these findings, corticosteroids have been investigated for use in CSDH management. Corticosteroids may prevent the continuing growth of subdural effusion because of their anti-inflammatory effects, as well as anti-angiogenic effects, which reduce fluid exudation and bleeding (Guénot, 2001; Santarius et al., 2008). The use of corticosteroids in the treatment of CSDH was first reported in 1968 (Bender and Christoff, 1974). Since then, an increasing number of studies have investigated the effects of corticosteroids on CSDH. Sun et al. (2005) reported that the routine use of adjuvant corticosteroids with surgery may lead to better outcomes compared to surgery alone. Chan et al. (2015) performed the first RCT on this topic and demonstrated that adjuvant corticosteroids with surgical drainage reduce the recurrence risk and reoperation rate.

The current study aimed to evaluate the efficacy and safety of adjuvant corticosteroids with surgery in the treatment of CSDH based on previous studies. The meta-analysis by Yao et al. (2017) suggested that overall dexamethasone (alone or adjuvant) may reduce recurrence rate, but does not improve poor outcome compared to surgical therapy. On the basis of the study by Yao et al. (2017) a meta-analysis was performed by Holl et al. (2019) which suggested that adjuvant corticosteroids with surgery reduce recurrence rate and mortality, however, Holl et al. (2019) mainly investigated the effectiveness between corticosteroids and surgery for the treatment of CSDH patients. Our study focused only on the comparison of effects between adjuvant corticosteroids with surgery and surgery alone. Our findings thus supplement those of Hutchinson et al. (2020), Mebberson et al. (2020), Lodewijkx et al. (2021), and Ng et al. (2021). Similar to previous studies, we found that adjuvant corticosteroids with surgery decrease the recurrence rate after surgery. However, in contrast to previous studies, our results showed that the clinical outcomes (all-cause mortality, functional outcome, and length of hospitalization) did not improve. In a recent multi-center RCT by Hutchinson et al. (2020) clinical outcomes and 6-month mortality rates were worse in the dexamethasone group than placebo, probably due to more adverse events in the dexamethasone group. Notably, Hutchinson et al. (2020) was included in the quantitative analysis due to the large sample size and that most patients (94%) underwent surgical evacuation. To explore the effects of study design on our results, we performed subgroup analyses, which demonstrated similar results across different study designs. There is concern regarding the systemic side effects and complications of corticosteroid therapy. We were unable to analyze the data on hyperglycemia and new-onset diabetes because of a lack of uniform diagnostic criteria and inadequate data. Hyperglycemia is common among corticosteroid-treated patients, but is easily controlled with rapid-acting insulin (Berghauser Pont et al., 2012). The pooled results showed that infection rates were not different between the adjuvant corticosteroid group and surgery alone group. In the recent studies by Hutchinson et al. (2020), Lodewijkx et al. (2021), and Ng et al. (2021) adverse events were significantly more common in the dexamethasone group. It is noteworthy that in these studies, long-term benefits of adjunctive corticosteroids were not observed, and adverse events were more common in the adjunctive corticosteroid group. Because CSDH recurrence is significantly related to the regrowth of residual CSDH during the early postoperative period, we focused on evaluating the short-term efficacy and safety of corticosteroids. Further research should evaluate the effects of corticosteroid dosing on the outcomes.

There were some limitations to the current review. First, most studies were conducted retrospectively, which may increase the risk of bias and reduce the robustness of the results. However, our subgroup analyses showed no significant differences in the results by study design. Second, there was significant variation between studies in the dosage and timing of administration, follow-up duration, initial severity of CSDH, and comorbidities, which may have introduced bias in the results. Finally, the number of included studies and participants was relatively small, which limited the statistical power of the results. Based on these limitations, larger, multi-center, high-quality RCTs are needed to validate our results.

## Conclusion

Adjuvant corticosteroids with surgery reduce the risk of recurrence of CDSH, but do not improve the all-cause mortality or functional outcome, as compared to surgery alone. These findings support the use of adjuvant corticosteroids with surgery for CSDH patients. Further high-quality RCTs are required to confirm the efficacy and safety of adjuvant corticosteroids in the treatment of CSDH patients.

## Data Availability Statement

The original contributions presented in the study are included in the article/Supplementary Material, further inquiries can be directed to the corresponding author/s.

## Author Contributions

MS: took responsibility for the integrity of the data and the accuracy of the data analysis. MS and L-FX: drafted the manuscript and performed statistical analysis. MS, L-FX, T-BZ, and Q-WT: made critical revision of the manuscript for important intellectual content. W-YZ: supervision. All authors: concept, design, analysis and interpretation of data.

## Conflict of Interest

The authors declare that the research was conducted in the absence of any commercial or financial relationships that could be construed as a potential conflict of interest.

## Publisher’s Note

All claims expressed in this article are solely those of the authors and do not necessarily represent those of their affiliated organizations, or those of the publisher, the editors and the reviewers. Any product that may be evaluated in this article, or claim that may be made by its manufacturer, is not guaranteed or endorsed by the publisher.

## Supplementary Material

Supplementary File 1 PRISMA 2009 Checklist.

Supplementary File 2 Free terms.

Supplementary File 3 Risk of bias graph of RCTs.
