# Supplementary material for: Adjuvant Corticosteroids With Surgery for Chronic Subdural Hematoma: A Systematic Review and Meta-Analysis
Source: Front Neurosci. 2021 Dec 8;15:786513. doi: 10.3389/fnins.2021.786513 (PMC8692773; doi:10.3389/fnins.2021.786513)
Supplement: Supplementary File 2 — Free terms. [file Data_Sheet_2.PDF]

Free terms：

(1) corticosteroid or steroid or prednisolone or prednisone or dexamethasone or cortisol or hydrocortisone or glucocorticoid or methylprednisolone

(2) chronic subdural hematoma or subdural hemorrhage or CSDH
