# Supplementary figures and images for: Adjuvant Corticosteroids With Surgery for Chronic Subdural Hematoma: A Systematic Review and Meta-Analysis
Source: Front Neurosci. 2021 Dec 8;15:786513. doi: 10.3389/fnins.2021.786513 (PMC8692773; doi:10.3389/fnins.2021.786513)

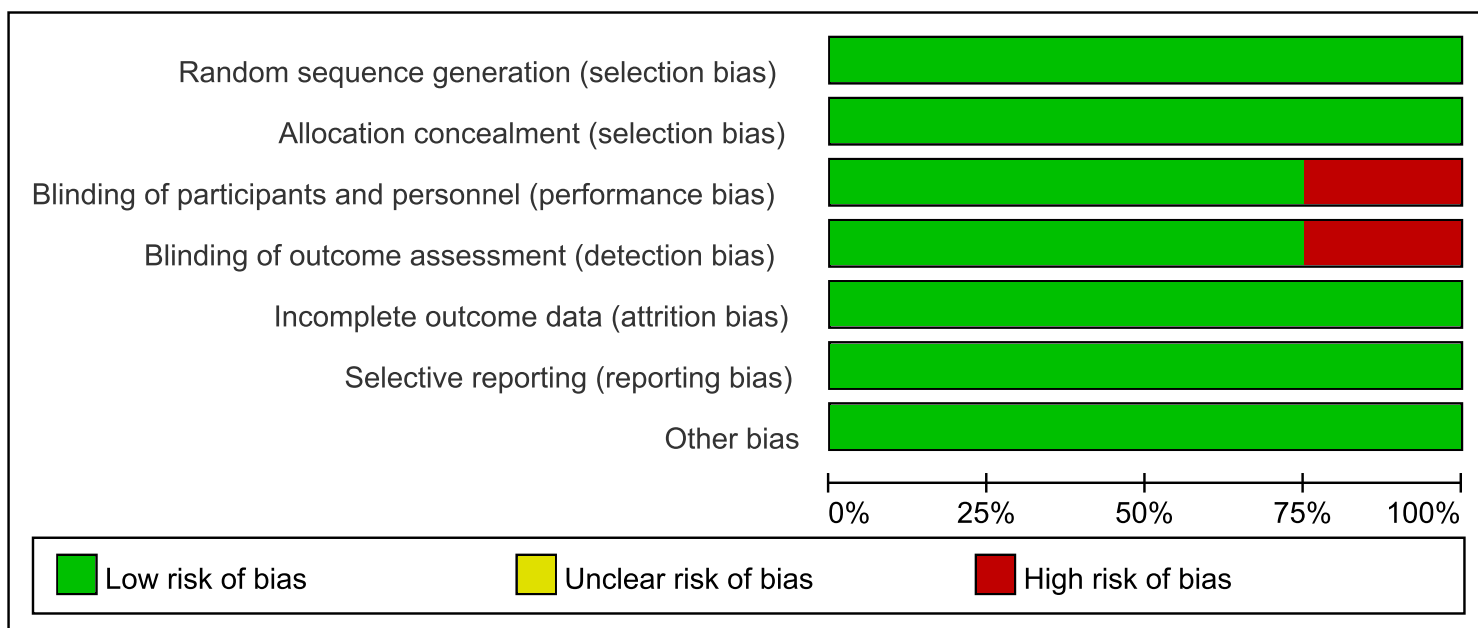

Supplement: Supplementary File 3 — Risk of bias graph of RCTs. [file Data_Sheet_3.PDF]
